# The non-linear correlation between the severity of alcohol consumption and depressive symptoms in the Chinese Wa ethnic minority

**DOI:** 10.3389/fpubh.2024.1430840

**Published:** 2024-10-30

**Authors:** Junjun Liu, Xiaotang Feng, Yang Liu, Libin Xiao, Ruixiang Tong, Yangchun Wang, Zhaomin Lu, Fengnan Jia, Xiaobin Zhang, Zhe Li, Xiangdong Du, Wanqiu Yang

**Affiliations:** ^1^Nanjing Meishan Hospital, Nanjing, China; ^2^Soochow University, Suzhou, China; ^3^Suzhou Guangji Hospital, The Affiliated Guangji Hospital of Soochow University, Suzhou, China; ^4^Nanjing Youan Hospital, Nanjing, China; ^5^School of Ethnology and Sociology, Yunnan University, Kunming, China

**Keywords:** correlation, alcohol use, depressive symptoms, ethnic minority, non-linear relationship

## Abstract

**Objective:**

The aim of this research was to examine the association between alcohol consumption and depressive symptoms in the Wa ethnic minority of China, a population where this relationship has been insufficiently explored.

**Methods:**

A cross-sectional analysis was conducted on a sample of 317 current drinkers from the Wa ethnic minority, a “direct fast-forward” group in Cangyuan County, between December 1, 2022, and February 28, 2023. Utilizing a multi-stage cluster sampling approach, participants were selected, each of whom exhibited an Alcohol Use Disorder Identification Test (AUDIT) score exceeding 0. Data were collected via face-to-face interviews employing a uniform questionnaire. Quantification of alcohol use was achieved through the application of the AUDIT, while the Patient Health Questionnaire (PHQ)-9 was employed to gauge depressive symptoms. The relationship between the severity of alcohol consumption and depressive symptoms was evaluated using a multivariable logistic regression model. Subsequently, potential non-linear associations were scrutinized through the application of a smoothing plot.

**Results:**

The study included 317 current drinkers (196 males [61.83%]; mean age 41.78 ± 12.91 years), of which 94 (29.65%) exhibited comorbid depressive symptoms. Multivariable logistic regression analysis, adjusting for confounders, revealed that higher AUDIT scores were significantly associated with an increased probability of depressive symptoms (OR = 1.09, 95% CI: 1.02–1.18, *p* = 0.008). The risk of depressive symptoms was notably greater in the group with alcohol dependent range in contrast the group at low-risk range (OR = 7.01, 95% CI: 1.66–29.62, *p* = 0.008). The smoothing plot indicated a J-shaped non-linear relationship with an inflection point at an AUDIT score of 15. To the left of this inflection point, no significant relationship was observed, whereas to the right, a positive correlation emerged (OR = 1.39, 95% CI: 1.11–1.74, *p* < 0.001).

**Conclusion:**

The findings reveal a non-linear relationship between alcohol consumption and the probability of depressive symptoms within the Wa ethnic minority in China, with implications for the development of nuanced and effective treatment strategies tailored to this population.

## 1 Introduction

Concomitant mental health challenges, particularly mood and anxiety disorders, frequently manifest alongside alcohol use disorders, presenting a complex clinical tableau (1). For instance, a meta-analysis of 22 population-based epidemiological surveys underscores a robust association between alcohol use disorders and major depressive episodes, whether assessed over a lifetime or within a 12-month period (2). Further research, examining a spectrum of alcohol consumption behaviors—ranging from binge drinking to problematic and at-risk drinking, to outright abuse—has elucidated a significant correlation with the emergence of depressive symptoms, as evidenced by a study conducted in an emergency department setting (3). Epidemiological data indicate the concurrent prevalence of depressive symptoms and alcohol use is prevalent, affecting between 15 and 28% of the general population (1, 4–6). Given this high prevalence, the interplay between depression and alcohol use disorder has been a focal point of investigation. Some studies have proposed a bidirectional causative mechanism, wherein increased alcohol consumption may elevate the risk for developing depression (7). This is further corroborated by findings that individuals with depression exhibit a heightened propensity for alcohol misuse. Indeed, Davis L. et al. report that nearly one-third of individuals diagnosed with major depressive disorder also meet the criteria for an alcohol use disorder (8). The global burden of these comorbid conditions is nontrivial; alcohol use disorder and major depressive disorder (MDD) rank among the top five contributors to disability-adjusted life years in developed nations (9). Comorbid depressive symptoms in individuals with alcohol use disorder portends a constellation of deleterious outcomes. These include elevated risk of suicide attempts (10), higher rates of relapse following abstinence (11), impaired overall functioning, and diminished life satisfaction (12).

Nevertheless, a compendium of research posits that alcohol consumption may serve as a palliative approach for mitigating distressing emotions and attenuating symptoms of compromised mental health (13–15). Supporting this notion, longitudinal investigations into the nexus between alcohol use and mental health outcomes provide strong evidence for a model wherein alcohol functions as a form of self-medication. In this model, deteriorating mental health emerges as a primary predictor for escalating alcohol consumption and may even perpetuate sustained heavy drinking (16). However, complicating this perspective, a body of evidence, indicative of a J-or U-shaped relationship, suggests that moderate alcohol consumption correlates with a reduced incidence of depression and enhanced mental well-being, in stark contrast to patterns observed in heavy drinkers and abstainers (17, 18). The task of fully elucidating or reconciling the disparate findings across these studies is formidable. This challenge is potentially attributable to heterogeneity in study cohorts, pre-existing physical health conditions (19), methodologies employed in assessing depression and alcohol use (20), among other variables. Furthermore, the extent to which these patterns of alcohol use and mental health concerns are consistent across different ethnic groups remains less defined.

Epidemiological investigations have delineated variances in alcohol consumption patterns across diverse racial and ethnic demographics (21). These studies reveal significant disparities. For instance, American Indians and Alaska Natives are reported to exhibit some of the highest prevalences of alcohol use disorder, with consumption levels that predispose these populations to an elevated burden of alcohol-related complications (22). In contrast, Asian Americans appear to experience a lower impact from such disorders. Beyond these ethnic differences, the literature further suggests that factors such as nativity and the degree of acculturation to American society exert a significant influence on drinking behaviors. The genesis of racial and ethnic disparities in alcohol consumption and the prevalence of alcohol use disorders is complex. It involves the intertwining of historical consumption trends with a spectrum of environmental, societal, and individual determinants (23), including persistent socioeconomic challenges. Given this complexity, there is an imperative for augmented research to elucidate facilitators and barriers to treatment access and support. Accordingly, this manuscript proceeds to expound upon prospective avenues for future inquiry.

China’s demographic landscape encompasses a rich tapestry of 55 officially recognized ethnic groups, many of which are numerically minor and reside in remote regions. Among these, a subset of groups, specifically the Wa, Bulang, Jingpo, Dulong, De’ang, Lisu, Jino, Nu, and Lahu, have undergone a remarkable sociocultural transition. Until the 1960s, these ethnic groups maintained traditional slash-and-burn farming lifestyles, used only native languages, and lived in isolated family-based social structures. Since then, particularly after 1994, the Chinese government’s poverty alleviation projects have rapidly transformed their economic situation. These groups have leapfrogged directly from a pre-modern existence characterized by hunting and shifting cultivation into a socialist framework with improved economy, education, and health. This dramatic transition, bypassing gradual modernization stages, characterizes them as “direct fast-forward” groups (24). Within these ethnic minorities, alcohol plays a pivotal role in social customs and festivities, with consumption deeply embedded in their cultural practices and a generally permissive stance towards drinking (25). The Wa people, in particular, present a unique case study for several reasons. Indigenous inhabitants of the Awa Mountains in Yunnan, proximal to international frontiers and Myanmar, and numbering approximately 350,000, the Wa people have experienced rapid socioeconomic changes due to their strategic location and recent integration into mainstream Chinese society. This transition has potentially influenced their traditional practices, including alcohol consumption patterns. The Wa culture places a significant cultural emphasis on alcohol, particularly in the context of hospitality, encapsulated by the adage, “without drinking, there is no etiquette.” The present study focuses on the Wa ethnic minority to address this gap and to provide insights into how traditional cultural practices surrounding alcohol interact with mental health in the context of rapid sociocultural transition. The present study seeks to elucidate the correlation between alcohol dependence and depressive symptoms among active alcohol consumers within the Wa ethnic minority in Yunnan Province, thereby contributing to a more nuanced understanding of these interrelations within this specific cultural context.

## 2 Methods

### 2.1 Ethical approval and consent process

The Institutional Review Board of Yunnan University (CRSS) granted ethical approval for the present study, with all participants providing informed consent in accordance with established protocols. The methodological approach adopted in this investigation was guided by the Strengthening the Reporting of Observational Studies in Epidemiology (STROBE) initiative. In this study, participation was entirely voluntary, and no monetary incentives, material rewards, or educational resources were offered to participants.

### 2.2 Sampling procedure

Employing a multi-stage cluster sampling method, a cross-sectional analysis was executed in Cangyuan County, situated within Yunnan Province, China, over the period from December 2022 to February 2023. The focus of the study was the Wa ethnic minority, a group historically isolated in remote, mountainous hamlets, maintaining a traditional lifestyle and utilizing their indigenous language until the year 1964. The sampling framework for this investigation was meticulously structured in four distinct phases, in alignment with the procedural standards of the Chinese Longitudinal Healthy Longevity Survey (CLHLS) (26). Initially, from the aggregate of 90 villages within Cangyuan County, a random selection of eight administrative villages was performed. Subsequently, within each administrative village, two natural villages were chosen, culminating in a total of 16 natural villages. In the third phase, a systematic sampling method was employed to select between 25 to 50 households from each natural community. Finally, the Kish Grid technique was utilized to randomly select one individual from each household for participation in the study (27).

### 2.3 Participant recruitment and screening

The following criteria were outlined for eligibility to participate in the study: (i) age from 18 to 69 years; (ii) residency in Cangyuan County for a minimum duration of 1 year; (iii) familial ties to the Wa ethnic minority; and (iv) active engagement in alcohol consumption. Exclusion criteria were established to ensure the integrity of the study’s outcomes, encompassing: (i) current pregnancy or lactation; (ii) a documented history of mental illness at any juncture; and (iii) the presence of significant comorbid physical conditions, including cancer, chronic infections, cerebral injuries, epilepsy, or cerebrovascular events. The study successfully recruited a cohort of 317 individuals, with an average age of 41.8 ± 12.9 years, comprising 196 males and 121 females. The reliability of the administered questionnaire was confirmed, with a validity rate reported at 97%.

### 2.4 Data collection process

Participant demographics and health-related behaviors were ascertained through structured interviews, encompassing age, sex, annual household income, body mass index (BMI), marital status, educational attainment, alcohol consumption frequency, and smoking status. Marital status was categorized as ‘single’ or ‘married’. Educational levels were bifurcated into ‘junior high school and below’ and ‘senior high school and above’. Alcohol consumption was stratified into five frequencies: ‘abstinent’, ‘monthly or less’, ‘two to four times per month’, ‘two to three times per week’, and ‘four or more times per week’. Smoking status was dichotomized into ‘current’ and ‘former’, the latter including both never-smokers and ex-smokers. Anthropometric data were systematically collected for each participant, with measurements of height recorded in meters (m) and weight in kilograms (kg). BMI, a crucial anthropometric indicator, was derived by applying the standard formula: BMI = weight (kg) / height^2 (m^2), enabling a standardized assessment of adiposity across participants.

### 2.5 Scale evaluation and quality control measures

The 10-item Alcohol Use Disorder Identification Test (AUDIT), a screening tool validated against DSM-IV criteria for alcohol use disorders, serves to discern individuals at elevated risk for developing problematic alcohol use within a forthcoming six-month period. The AUDIT scoring system, which spans from 0 to 40 and has undergone validation and standardization within the Chinese demographic, correlates positively with the severity of alcohol dependence. Participants with an AUDIT score of zero were deemed abstainers and consequently excluded from the study. The remaining participants were stratified into three risk categories for alcohol consumption: ‘low-risk range’ for scores ranging from 1 to 7, ‘hazardous or harmful rage’ for scores between 8 and 14, and ‘alcohol dependent range’ for scores of 15 or above (28).

Depressive symptomatology was quantified utilizing the Patient Health Questionnaire (PHQ-9), with a dichotomous classification of symptoms as ‘present’ for scores equal to or exceeding 5, and ‘absent’ for scores below this threshold (29). The PHQ-9 suicide item provided a succinct metric for gauging the prevalence of suicidal ideation. Specifically, this item probes the frequency of individuals’ contemplations of death or self-injury within the antecedent fortnight, encompassing both passive death wishes and active self-harm considerations.

The assessment of anxiety symptoms was conducted using the Generalized Anxiety Disorder 7-item (GAD-7) scale, which encompasses a comprehensive suite of seven anxiety-related questions. The GAD-7 scale yields a cumulative score, with a potential range from 0 to 21, serving as an index of the aggregate severity of anxiety symptoms (30). Within the context of the present investigation, a GAD-7 score of 5 or above was indicative of the presence of at least mild anxiety symptoms.

The Cronbach’s alpha coefficients for the AUDIT, PHQ-9, and GAD-7 in this study were 0.89, 0.90, and 0.94, respectively. To accommodate the predominantly low educational attainment of the participants, a significant proportion of whom were either illiterate or possessed limited formal education, data collection was facilitated through in-person interviews. The interviews were carried out by personnel who underwent comprehensive training. Trained psychiatrists with relevant expertise administered the AUDIT, the PHQ-9, and the GAD-7 scale. To ensure uniformity and dependability in the data acquisition process, these instruments were applied repeatedly using standardized protocols. The inter-rater reliability of these assessments was rigorously maintained, with a correlation coefficient exceeding 0.8, thereby affirming the consistency of the evaluative measures employed.

### 2.6 Statistical analysis

R (version 4.3.0) and EmpowerStats (version 4.2.0, accessible at http://www.empowerstats.com/cn/) were used for statistical analyses. A two-tailed *p*-value of less than 0.05 was recognized as the threshold for statistical significance. While continuous variables were reported using means and standard deviations (SD), categorical variables were shown as counts and percentages. The study population was stratified into low-risk range group, hazardous or harmful rage group, and alcohol dependent range group. One-way analysis of variance (ANOVA) was used to compare continuous data between groups, while the Chi-square test was used to evaluate categorical variables. Logistic regression analyses were conducted to explore the linear relationship between AUDIT scores and symptoms of depression. Within these models, AUDIT scores were treated both as a categorical variable and as a continuous measure, delineated by varying levels of alcohol consumption. The variance inflation factor (VIF) was computed in order to address potential multicollinearity among independent variables, with a VIF exceeding five indicating significant multicollinearity, leading to the exclusion of the affected variables from the final model. Potential confounding variables were included in the multivariable model if they exhibited a *p*-value of less than 0.10 in univariable analyses or altered the estimated effect of AUDIT scores on depressive symptomatology by more than 10% (31). Three logistic regression analysis models were built to elucidate the relationship between AUDIT scores and depressive symptoms: an unadjusted model, Model I adjusted for sex and age, and Model II further adjusted for variables including the frequency of alcohol intake, suicidal ideation, and anxiety symptoms. When the relationship between AUDIT scores and depressive symptoms was found to be non-linear, a smoothing plot was employed to guide the development of a two-piecewise linear regression model that relied on the Generalized Estimating Equation (GEE) method to ascertain the threshold effect.

## 3 Results

### 3.1 Participant demographics at baseline

In the present study, the Wa ethnic minority was represented by 317 individuals. With a standard deviation of 12.91 years, the individuals’ mean age was 41.78 years. The cohort comprised 38.17% females (*n* = 121) and 61.83% males (*n* = 196). Depressive symptoms were observed in 29.65% of the participants (*n* = 94), with prevalence rates of 31.58% (*n* = 42/133) in the low-risk range group, 24.70% (*n* = 41/166) in the hazardous or harmful rage group, and 61.11% (*n* = 11/18) in the alcohol dependent range group. Baseline characteristics of the participants, stratified by AUDIT scores, are detailed in Table 1. Significant correlations were identified between AUDIT scores and several variables, including marital status, frequency of alcohol consumption, and suicidal ideation, with all associations yielding *p*-values less than 0.05.

**Table 1:** Baseline characteristics of participants.

Variables	Total	Low-risk range	Hazardous or harmful range	Alcohol dependent range	*P*-value
*N*	317	133	166	18	
Age (years)	41.78 ± 12.91	41.93 ± 11.86	41.35 ± 13.65	44.60 ± 13.68	0.589
Annual household income (Yuan)	48380.95 ± 42471.85	45077.62 ± 36376.09	52713.80 ± 47959.94	32830.44 ± 20157.91	0.084
BMI (kg/m^2^)	24.17 ± 3.70	24.18 ± 3.84	24.03 ± 3.66	25.46 ± 2.89	0.302
AUDIT score	9.08 ± 5.63	4.10 ± 2.19	11.58 ± 3.08	22.83 ± 2.15	<0.001
Gender					0.344
Male	196 (61.83%)	79 (59.40%)	108 (65.06%)	9 (50.00%)	
Female	121 (38.17%)	54 (40.60%)	58 (34.94%)	9 (50.00%)	
Education					0.394
Junior high and below	276 (87.07%)	118 (88.72%)	141 (84.94%)	17 (94.44%)	
Senior high and above	41 (12.93%)	15 (11.28%)	25 (15.06%)	1 (5.56%)	
Ethnic group					0.229
Han	4 (1.26%)	1 (0.75%)	2 (1.20%)	1 (5.56%)	
Wa	313 (98.74%)	132 (99.25%)	164 (98.80%)	17 (94.44%)	
Marital status					0.005
Single	73 (23.03%)	19 (14.29%)	50 (30.12%)	4 (22.22%)	
Married	244 (76.97%)	114 (85.71%)	116 (69.88%)	14 (77.78%)	
Current smoking					0.482
No	200 (63.09%)	84 (63.16%)	107 (64.46%)	9 (50.00%)	
Yes	117 (36.91%)	49 (36.84%)	59 (35.54%)	9 (50.00%)	
The frequency of drinking alcohol					<0.001
<= 1 time per month	111 (35.02%)	79 (59.40%)	32 (19.28%)	0 (0.00%)	
2–4 times per month	72 (22.71%)	29 (21.80%)	39 (23.49%)	4 (22.22%)	
2–3 times per week	65 (20.50%)	17 (12.78%)	46 (27.71%)	2 (11.11%)	
> = 4 times per week	69 (21.77%)	8 (6.02%)	49 (29.52%)	12 (66.67%)	
Suicidal ideation					0.008
No	306 (96.53%)	125 (93.98%)	165 (99.40%)	16 (88.89%)	
Yes	11 (3.47%)	8 (6.02%)	1 (0.60%)	2 (11.11%)	
Comorbid depressive symptoms					0.005
No	223 (70.35%)	91 (68.42%)	125 (75.30%)	7 (38.89%)	
Yes	94 (29.65%)	42 (31.58%)	41 (24.70%)	11 (61.11%)	
Comorbid anxiety symptoms					0.089
No	256 (80.76%)	110 (82.71%)	135 (81.33%)	11 (61.11%)	
Yes	61 (19.24%)	23 (17.29%)	31 (18.67%)	7 (38.89%)	

AUDIT: the alcohol use disorder identification test; low-risk range: AUDIT score 1–7; hazardous or harmful rage: AUDIT score 8–14; alcohol dependent range: AUDIT score > 14.

### 3.2 Univariate analysis of factors linked to depressive symptoms

The results of the univariate analysis are presented in Table 2. The analysis revealed statistically significant associations between depressive symptoms and several variables. With an OR of 6.82 and a 95% CI ranging from 1.77 to 26.32, it was noteworthy that there was a strong positive connection between depressed symptoms and suicidal ideation. The probability of experiencing depressive symptoms was also found to be slightly but significantly elevated in relation to an increase in the AUDIT score (OR = 1.04, 95% CI: 1.00 to 1.09). Additionally, the presence of anxiety symptoms was strongly correlated with depressive symptoms, as indicated by an OR of 10.66 (95% CI: 5.63 to 20.21). All aforementioned associations were statistically significant with *p*-values below 0.05. Conversely, no significant correlations were observed between depressive symptoms and variables such as the frequency of alcohol consumption, age, gender, marital status, annual household income, BMI, level of education, ethnic group, and current smoking status, with all p-values exceeding 0.05.

**Table 2:** Univariate analysis for depressive symptoms.

Covariate	OR	95% CI	*P*-value
*N* = 317			
Age (years)	1.00	0.98, 1.02	0.865
Annual household income (Yuan)	1.00	1.00, 1.00	0.838
BMI (kg/m^2^)	1.04	0.97, 1.07	0.262
AUDIT score	1.04	1.00, 1.09	0.045
Gender			
Male	1.0 (Reference)		
Female	0.83	0.50, 1.37	0.466
Education			
Junior high and below	1.0 (Reference)		
Senior high and above	0.79	0.47, 1.32	0.369
Ethnic group			
Han	1.0 (Reference)		
Wa	0.42	0.06, 3.00	0.385
Marital status			
Single	1.0 (Reference)		
Married	0.97	0.55, 1.72	0.918
Current smoking			
No	1.0 (Reference)		
Yes	0.96	0.58, 1.58	0.860
The frequency of drinking alcohol			
<= 1 time/month	1.0 (Reference)		
2–4 times/month	1.23	0.65, 2.34	0.519
2–3 times/week	1.54	0.81, 2.95	0.189
> = 4 times/week	0.57	0.28, 1.19	0.135
Suicidal ideation			
No	1.0 (Reference)		
Yes	6.82	1.77, 26.32	0.005
Comorbid anxiety symptoms			
No	1.0 (Reference)		
Yes	10.66	5.63, 20.21	<0.001

AUDIT, the alcohol use disorder identification test; OR, odds ratio; CI, confidence interval.

### 3.3 Relationship between alcohol use and depressive symptoms

Table 3 illustrates the substantial positive correlation that was identified between high AUDIT scores and depressed symptoms after thorough correction for confounding variables. Individuals with higher AUDIT scores demonstrated an increased odds ratio (OR) of 1.09 for depressive symptoms, with a 95% confidence interval (CI) spanning from 1.02 to 1.16, and the association was statistically significant (*p* = 0.008). Further analysis revealed that participants with AUDIT scores in the range of 15 to 40 were substantially more probable to report depressive symptoms (OR = 7.01, 95% CI: 1.66 to 29.62) than those with lower AUDIT scores of 1 to 7, maintaining statistical significance (*p* = 0.008) after adjusting for potential confounders.

**Table 3:** Relationship between alcohol dependence score and depressive symptoms in different models.

Variable	N	Unadjusted Model		Model I		Model II	
		OR (95%CI)	P-value	OR (95%CI)	P-value	OR (95%CI)	P-value
AUDIT score	317	1.04 (1.00, 1.09)	0.045	1.04 (1.00, 1.09)	0.046	1.09 (1.02, 1.16)	0.008
AUDIT score categorical							
1–7	133	1.0 (Reference)		1.0 (Reference)		1.0 (Reference)	
8–4	166	0.71 (0.43, 1.18)	0.188	0.70 (0.42, 1.17)	0.170	0.81 (0.42, 1.56)	0.527
15–40	18	3.40 (1.23, 9.40)	0.018	3.51 (1.26, 9.73)	0.016	7.01 (1.66, 29.62)	0.008
P for trend		0.516		0.523		0.271	

AUDIT, the alcohol use disorder identification test; OR, odds ratio; CI, confidence interval; Unadjusted Model adjusted for none; Model I adjusted for age, sex; Model II adjusted for age, sex, the frequency of drinking alcohol, suicidal ideation, and anxiety symptoms.

### 3.4 Examination of non-linear associations via generalized additive models

**Figure 1:**
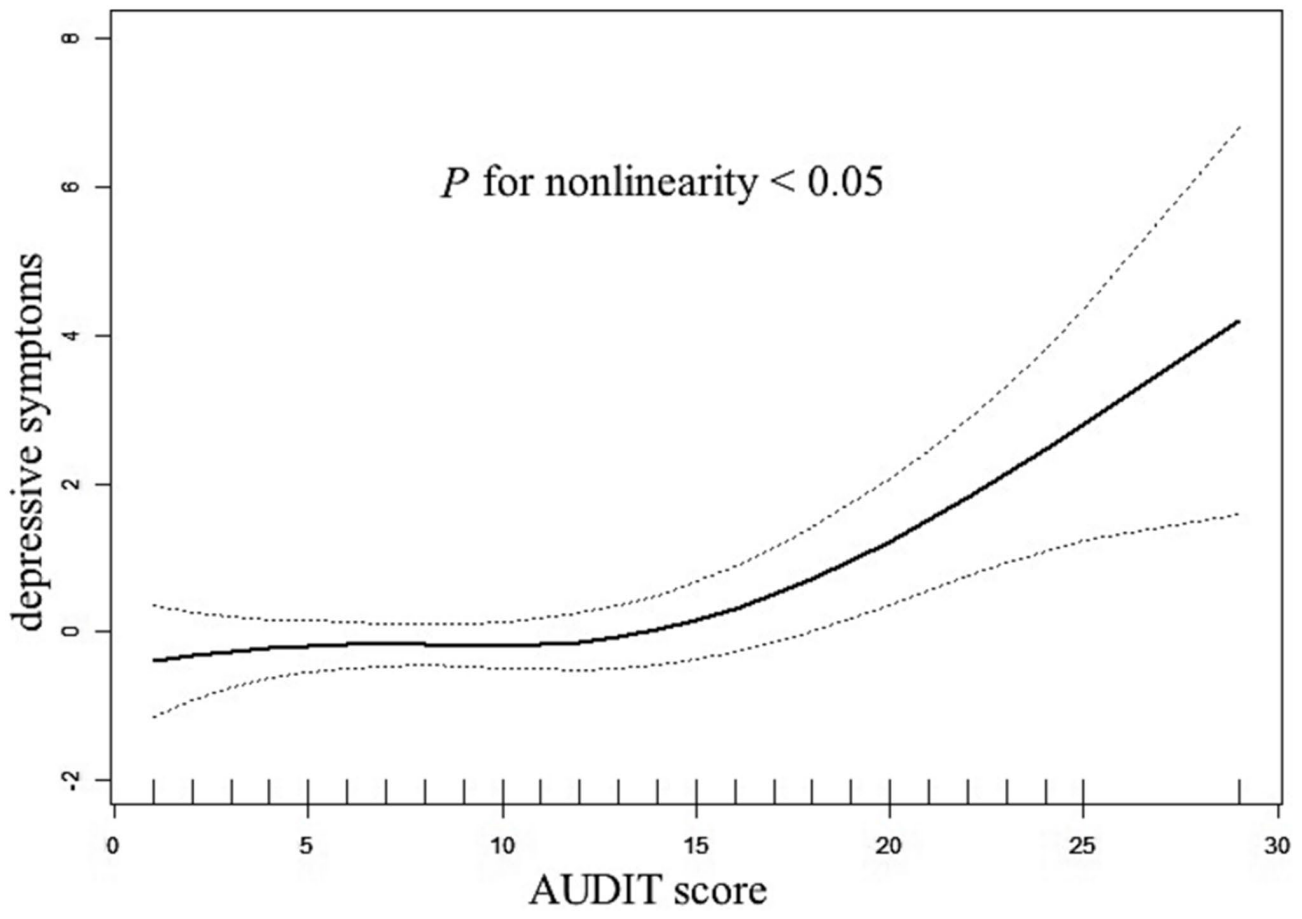
The relationship between AUDIT scores and depressive symptoms. A nonlinear relationship between AUDIT scores and the probability of depressive symptoms was observed after adjusting for age, sex, the frequency of alcohol intake, suicidal ideation, and anxiety symptoms (P for non-linearity <0.05).

The application of generalized additive models revealed a non-linear relationship between AUDIT scores and the manifestation of depressive symptoms, with a significant test for non-linearity (*p* < 0.05), as depicted in Figure 1. At a AUDIT score of 15, a two-segment logistic regression model pinpointed an inflection point. Above this cutoff, the odds of reporting depressive symptoms increased by 39% with each additional AUDIT score increase (OR = 1.39, 95% CI: 1.16 to 1.68, *p* < 0.001). Conversely, as presented in Table 4, AUDIT scores below the inflection point did not exhibit a statistically significant correlation with depressive symptoms (OR = 1.00, 95% CI: 0.92 to 1.09, *p* = 0.934). Bootstrap resampling techniques were employed to ascertain 95% confidence intervals for the inflection point, which ranged between AUDIT scores of 13 and 19. Within the study cohort, 52 participants recorded AUDIT scores of 15 or higher, while 265 participants had scores below this threshold.

**Table 4:** The results of two-piecewise logistic regression model.

Inflection point of AUDIT score	OR	95%CI	*P*-value
Inflection point	15	13 to 19	
< 15 slope 1 (*n* = 265)	1.00	0.92 to 1.09	0.934
≥ 15 slope 2 (n = 52)	1.39	1.16 to 1.68	<0.001
slope 2 – slope 1	1.39	1.11 to 1.74	0.005
predicted at 15	−1.21	−1.74 to-0.68	
Log likelihood ratio test			0.003

Effect: Depressive symptoms; Cause: AUDIT score; adjusted for age, sex, age, sex, the frequency of drinking alcohol, suicidal ideation, and anxiety symptoms. Slope 1 and slope 2 are the slope coefficients for the segment before and after the inflection point.

## 4 Discussion

In a pioneering investigation into the interplay between alcohol consumption and depressive symptoms within the Wa ethnic minority of China, our study presents several novel findings. Initially, we observed that a significant proportion, 29.65% (94 out of 317), of the drinking demographic in the Wa community exhibited symptoms indicative of depression. Subsequent analysis utilizing a fully adjusted regression model revealed a positive correlation between AUDIT score and the prevalence of depressive symptoms. Specifically, individuals classified within the alcohol-dependent category were found to be at a 7.01-fold greater risk of developing depressive symptoms compared to their counterparts engaging in low-risk drinking behaviors. Furthermore, our research identified a J-shaped non-linear association between AUDIT scores and depressive symptoms, pinpointing an inflection point at a score of 15. With every additional point that the AUDIT score increases, there is a noticeable 39% rise in the likelihood of depressive symptoms to the right of this inflection point. Conversely, the correlation was not statistically significant to the left of this inflection point. The findings of this study contribute a nuanced understanding of the relationship between alcohol consumption and mental health, taking into account additional influencing factors. This research underscores the complex nature of alcohol-related health risks and their impact on mental well-being within this specific ethnic minority, offering valuable insights for public health interventions and policy formulation.

The global public health landscape is increasingly recognizing the ramifications of alcohol consumption, with particular emphasis on misuse, dependency, and associated mental health disorders (32). Epidemiological evidence suggests that the patterns of alcohol use, the prevalence of related complications, and the engagement with treatment services exhibit considerable heterogeneity across different racial and ethnic groups (22). However, the incidence of depressive symptoms among the drinking populations of China’s rapidly modernizing ethnic minorities remains underexplored. Our investigation contributes to this knowledge gap, revealing that 29.65% of individuals who consume alcohol within this demographic concurrently exhibit depressive symptoms. This prevalence aligns with international data, such as the 24.7% incidence of depressive symptoms among Serbian patients with hazardous alcohol use, as determined by the Beck Depression Inventory with a threshold of 21 points (33). Comparable findings emerge from the National Comorbidity Survey Replication, which reports a 21.0% co-occurrence of severe depression and alcohol dependence (34), and from Caetano’s study in Puerto Rico, where 23% of individuals with alcohol use disorders were affected by major depression (35). Contrastingly, Huang et al. report a markedly higher prevalence of major depression at 48.9% among Chinese Han psychiatric patients diagnosed with alcohol dependency (1). Similarly, research by Odlaug et al. (36) across eight European countries found that 43.1% of 2,979 individuals with alcohol dependence also suffered from depression. The discrepancies observed across these studies may be attributable to variations in the diagnostic criteria and assessment tools used to evaluate alcohol consumption and depressive symptoms. It is noteworthy that patients with alcohol dependence in psychiatric settings are more likely to exhibit severe dependency and depressive symptoms compared to the general population.

Emerging evidence underscores a robust association between alcohol use disorders (AUD) and an elevated risk of major depressive disorder (MDD). A significant linkage between MDD and both 12-month and lifetime AUD prevalence has been substantiated by the National Epidemiologic Survey on Alcohol and Related Conditions III, with odds ratios of 4.24 (95% CI 3.51–5.13) observed across the spectrum of AUD severity (37). Complementary findings from a cross-sectional analysis in a Los Angeles emergency department by Bazargan-Hejazi et al. (3) indicate that various drinking behaviors, including binge drinking, alcohol abuse, and at-risk consumption, correlate with the manifestation of depressive symptoms, as measured by multiple alcohol consumption metrics (AUDIT, DSM IV-Abuse, and binge drinking). Boden’s synthesis of the literature posits a causal nexus between MDD and AUD, suggesting that the presence of an AUD doubles the risk of a major depressive episode (7). Despite the well-documented comorbidity of alcohol dependence and depression, the underlying mechanisms remain a subject of scholarly debate. Several hypotheses have been proposed to elucidate the co-occurrence of these conditions. Firstly, alcohol’s pharmacological properties as a central nervous system depressant may potentiate depressive symptoms (33). Secondly, individuals experiencing depression might concurrently develop AUD as a coping mechanism in response to adverse life events, such as bereavement, unemployment, financial strain, or significant familial stress (38, 39). Thirdly, the self-medication hypothesis suggests that individuals with depression may consume alcohol to alleviate their symptoms, potentially leading to dependence (40). Additionally, the Wa ethnic minority, predominantly rural dwellers, may experience a disproportionate burden of alcohol abuse and mental health challenges, potentially exacerbated by socioeconomic disadvantages, including limited financial resources and educational attainment (41). A further dimension to consider is the neuroimmunological changes associated with alcoholism and depression, particularly the activation of immune responses that may influence negative affect, drug-seeking behavior, and behavioral control through neuroimmune gene activation in limbic regions (42, 43). Lastly, genetic studies have begun to unravel a shared genetic susceptibility between depression and alcoholism. For instance, Muench et al. (44) identified that the MDD risk allele rs10514299 is predictive of the reward mechanism in alcohol dependency, while Zhou et al. (45) reported an association between SEMA3A variants and severe depression concurrent with alcohol dependence.

There seems to be a saturation effect and a J-shaped non-linear correlation between alcohol use and depression symptoms. Further investigation into the dose–response relationship between alcohol intake and depressive symptomatology reveals a critical inflection point; individuals presenting with an AUDIT score in excess of 15 demonstrate a marked increase in the probability of experiencing depressive states. This finding is only partially congruent with prior studies that have implicated high-risk drinking behaviors, such as binge and heavy drinking, in the development of alcohol-related problems (46, 47). A recent meta-analysis reaffirms that heavy drinkers face an elevated risk of depression, while light to moderate alcohol intake may confer a reduced risk of depressive disorders compared to abstention (48). Additional research suggests that individuals who engage in regular low-to-moderate alcohol consumption are less likely to report depression than those who abstain entirely (18). Potential biological mechanisms underlying the protective effects of moderate alcohol consumption include dopaminergic and GABAergic pathways (49, 50), as well as increased levels of brain-derived neurotrophic factors and decreased inflammatory biomarkers, both of which have been implicated in depression. However, it is important to acknowledge that in certain cultural contexts, low-to-moderate alcohol consumption may be indicative of better social integration compared to abstention, a factor known to protect against depression (51). When interpreting these findings, it’s crucial to consider the unique cultural and environmental factors of the Wa ethnic minority. The Wa people have a long-standing tradition of alcohol use in social contexts, encapsulated by the saying ‘without drinking, there is no etiquette.’ This cultural norm, combined with their rapid transition from traditional to modern lifestyles, may influence both alcohol consumption patterns and mental health outcomes. Additionally, the geographical isolation of many Wa communities and extremely low levels of health literacy (0.89%) may limit access to health information and mental health resources (24). These findings of the present study warrant cautious interpretation due to the absence of detailed information on participants’ patterns of alcohol consumption, including the specific quantity, frequency, and duration of use. Understanding of the connection between alcohol consumption and depression may be significantly influenced by this kind of information. It is recommended that routine population-based alcohol screenings be implemented in primary care settings to identify individuals engaging in harmful alcohol use who may benefit from targeted brief alcohol interventions.

This study represents a pioneering investigation into the association between alcohol consumption and depressive symptoms within the Wa ethnic group in China, employing a robust methodological approach that includes both linear and nonlinear regression analyses and accounts for a wide array of potential confounders. Notably, this is the first study of its kind to focus on this particular ethnic group, offering novel insights into the public health challenges faced by the Wa community. Despite the strengths of this research, there are several limitations that offer potential areas for future lines of research. The cross-sectional design of this study is limited to identifying correlations between alcohol use and depressive symptoms; it does not establish a causal relationship between the two. Therefore, further longitudinal studies are necessary to elucidate the temporal relationship between these factors. Furthermore, the use of self-reported data raises the risk of recollection bias and the impact of social desirability on participant answers. Although the instruments used in this study are validated screening tools, they are not diagnostic measures; consequently, further research is necessary to establish whether participants meet clinical thresholds for mental health disorders. The focus on the Wa ethnic minority in Cangyuan County, Yunnan Province, underscores the importance of extending this research to diverse populations to confirm the generalizability of the findings. Future research should explore the effectiveness of culturally tailored interventions for co-occurring alcohol use and depressive symptoms in the Wa community. This could include evaluating traditional healing practices alongside modern therapeutic approaches, potentially leading to more effective and culturally acceptable treatment strategies. In addition, only the AUDIT scores and the frequency of alcohol consumption of drinkers were collected; no data was collected on the types, patterns, and amounts of alcohol consumed. Moreover, while the study controlled for a range of confounding variables, it did not encompass all possible factors, such as biological markers, personality traits, and social status, which could significantly affect the relationship between alcohol use and depressive symptoms. Future investigations should aim to incorporate a wider range of potential confounding factors to elucidate the underlying pathophysiological mechanisms driving the observed association.

## 5 Conclusion

This study reveals a high prevalence (29.65%) of comorbid depressive symptoms among active alcohol consumers in the Wa ethnic minority of China, with a significant positive correlation between AUDIT scores and depressive symptoms. We identified a non-linear J-shaped relationship, with an inflection point at an AUDIT score of 15, above which each additional point increase corresponded to a 39% higher likelihood of depressive symptoms. Individuals in the alcohol-dependent category faced a 7.01-fold greater risk of developing depressive symptoms compared to low-risk drinkers. These findings underscore the complex interplay between alcohol consumption and mental health in this rapidly modernizing ethnic minority population, highlighting the urgent need for culturally sensitive interventions and policies that extend beyond healthcare to address the dual burden of alcohol misuse and depression. Our results provide valuable insights for public health strategies and emphasize the importance of tailored approaches in both research and interventions targeting alcohol use and mental health in the Wa population and potentially other ethnic minority groups undergoing rapid sociocultural transitions.

## Data Availability

The raw data supporting the conclusions of this article will be made available by the authors, without undue reservation.
